# Reactive oxygen metabolism in the proliferation of Korean pine embryogenic callus cells promoted by exogenous GSH

**DOI:** 10.1038/s41598-023-28387-5

**Published:** 2023-02-08

**Authors:** Fang Gao, Ruirui Wang, Yujie Shi, Hailong Shen, Ling Yang

**Affiliations:** 1 State Key Laboratory of Tree Genetics and Breeding, School of Forestry, Northeast Forestry University, Harbin, 150040 People’s Republic of China; 2 Institute of Biotechnology, Jilin Provincial Academy of Forestry Sciences, Changchun, 130033 People’s Republic of China; 3 State Forestry and Grassland Administration Engineering Technology Research Center of Korean Pine, Harbin, 150040 People’s Republic of China

**Keywords:** Plant biotechnology, Embryogenesis

## Abstract

Exogenous glutathione (GSH) promotes the proliferation of embryogenic callus (EC) cells in Korean pine in the course of somatic embryogenesis, and reactive oxygen species (ROS) may play an important role in regulating the proliferation of EC cells by exogenous GSH. However, the concrete metabolic response of ROS is unclear. In this study, two cell lines of Korean pine with high proliferative potential 001#-001 (F, Fast proliferative potential cell line is abbreviated as F) and low proliferative potential 001#-010 (S, Slow proliferative potential cell line is abbreviated as S) were used as test materials. The responses of ROS-related enzymes and substances to exogenous GSH and L-Buthionine-sulfoximine (BSO) were investigated in EC cells. The results showed that the exogenous addition of GSH increased the number of early somatic embryogenesis (SEs) in EC cells of both F and S cell lines, decreased the amount of cell death in both cell lines. Exogenous addition of GSH promoted cell division in both cell lines, increased intracellular superoxide dismutase (SOD) and catalase (CAT) activities, inhibited intracellular hydrogen peroxide (H_2_O_2_), malondialdehyde (MDA) and nitric oxide (NO) production, and increased NO/ROS ratio. In conclusion, the exogenous GSH promoting the proliferation of Korean pine EC cells, the activity of intracellular antioxidant enzymes was enhanced, the ROS level was reduced, and the resistance of cells to stress was enhanced.

## Introduction

The somatic embryogenesis (SE) technique enables the rapid propagation of high quality and quantity seedlings^1^, which is the most promising method of asexual reproduction. The embryogenic callus (EC) is the basis for large-scale SE, an important material for genetic transformation, and an ideal model for studying totipotency and single-cell differentiation. Therefore, EC in good condition and high proliferation rate during SE are essential. Korean pine (*Pinus koraiensis* Sieb. et Zucc.) is a dominant tree species of the mixed broad-leaved Korean pine forest in the temperate zone. It is also an important high-quality and valuable timber tree species and economic forest tree species of nut in China and East Asia. It has essential ecological, economic, and social value. Although Korean pine SE technology systems have been established^2–5^, problems such as low induction rate of EC, low proliferation rate, and poor growth of the induced EC affect further differentiation and maturation.

Reactive oxygen species (ROS) are chemically active oxygen-containing atoms or atomic groups, such as superoxide anion radical (O_2_^·−^), hydrogen peroxide (H_2_O_2_), and hydroxyl radical (^·^OH)^6^. During evolution, plants have formed two kinds of protective systems, enzymatic and non-enzymatic, which give them the ability to remove large amounts of reactive oxygen species (ROS) and prevent or alleviate the damage caused by ROS under conditions of adverse stress. Glutathione (GSH), a small molecular peptide composed of amino acids, is an important antioxidant and ROS scavenger in organisms, which has an important influence on intracellular ROS metabolism and protects cells from oxidative damage. The presence of GSH in plant cells^7–9^, giving the plant the ability to handle high levels of H_2_O_2_ production and a complex network of ROS reactions while maintaining redox homeostasis. Studies have shown that exogenous GSH can promote plant cell proliferation^10,11^. In Korean pine EC proliferation, the exogenous addition of GSH promoted EC proliferation in cell lines with different proliferative potential, with exogenous GSH treatment the proliferation of fresh weight reached the peak from day 7 to 14 (proliferation was 0.736 g, 19.5% higher than that of the control (CK)). ROS may play an important role in the regulation of EC proliferation by exogenous GSH, but the specific metabolic response pattern to it is unclear^12^. ROS are closely related to cell proliferation and differentiation^13–15^. EC proliferation is the result of the dynamic balance between cell division and differentiation. Therefore, the promoting effect of GSH on EC proliferation may be related to ROS. To understand the role of ROS in the regulation of EC proliferation by exogenous GSH and ROS metabolism response pattern, this study increases the GSH content of Korean pine EC cells by adding exogenous GSH while decreases it by adding exogenous l-Buthionine-sulfoximine (BSO) to investigate the dynamic change of the effects of exogenous GSH and BSO on nitric oxide (NO), hydrogen peroxide (H_2_O_2_), catalase (CAT), superoxide dismutase (SOD), cell death, cell division ratio, and malondialdehyde (MDA) in Korean pine EC cells, and to reveal the change pattern of ROS metabolism in the proliferation of Korean pine EC cells promoted by exogenous GSH.

## Results

### Effects of exogenous GSH and BSO on EC cell morphology and structure

**Figure 1:**
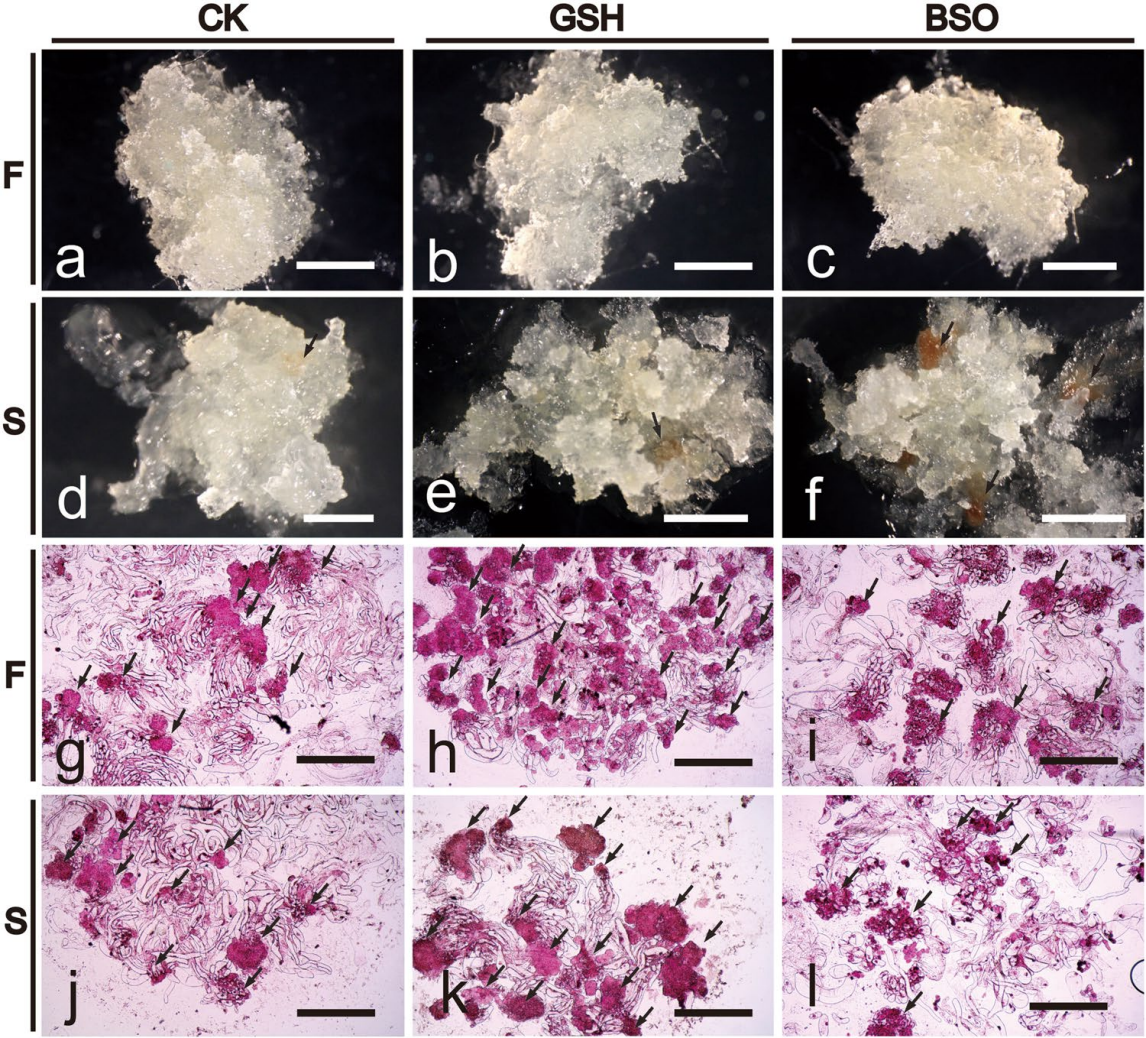
Effects of exogenous GSH and BSO on EC cell morphology of two Korean pine cell lines. (**a**) F-CK treatment; (**b**) F-GSH treatment; (**c**) F-BSO treatment; (**d**) S-CK treatment; (**e**) S-GSH treatment; (**f**) S-BSO treatment. (**a**–**f**) bars = 1 cm. (**g**) F-CK internal structure; (**h**) F-GSH internal structure; (**i**) F-BSO internal structure; (**j**) S-CK internal structure; (**k**) S-GSH internal structure; (**l**) S-BSO internal structure. (**g**–**h**) bars = 200 μm.

The effects of exogenous GSH and BSO on the morphology of EC cells of the F cell line was not significant (Fig. 1a–c), but those on the morphology of EC cells of the S cell line was greater (Fig. 1d–f). Compared with S-CK (Fig. 1d), the EC cells were opalescent and transparent after S-GSH treatment (Fig. 1e). S-BSO treatment increased the internal browning spots, and EC cells were opalescent and transparent accompanied by more browning spots (Fig. 1f). The exogenous addition of GSH and BSO had a greater effect on the internal structure of F and S cell lines (Fig. 1g–l). The exogenous addition of GSH stimulates the production of SE (Fig. 1h,k), which are more numerous but smaller than SE obtained using BSO (Fig. 1i,l).

**Figure 2:**
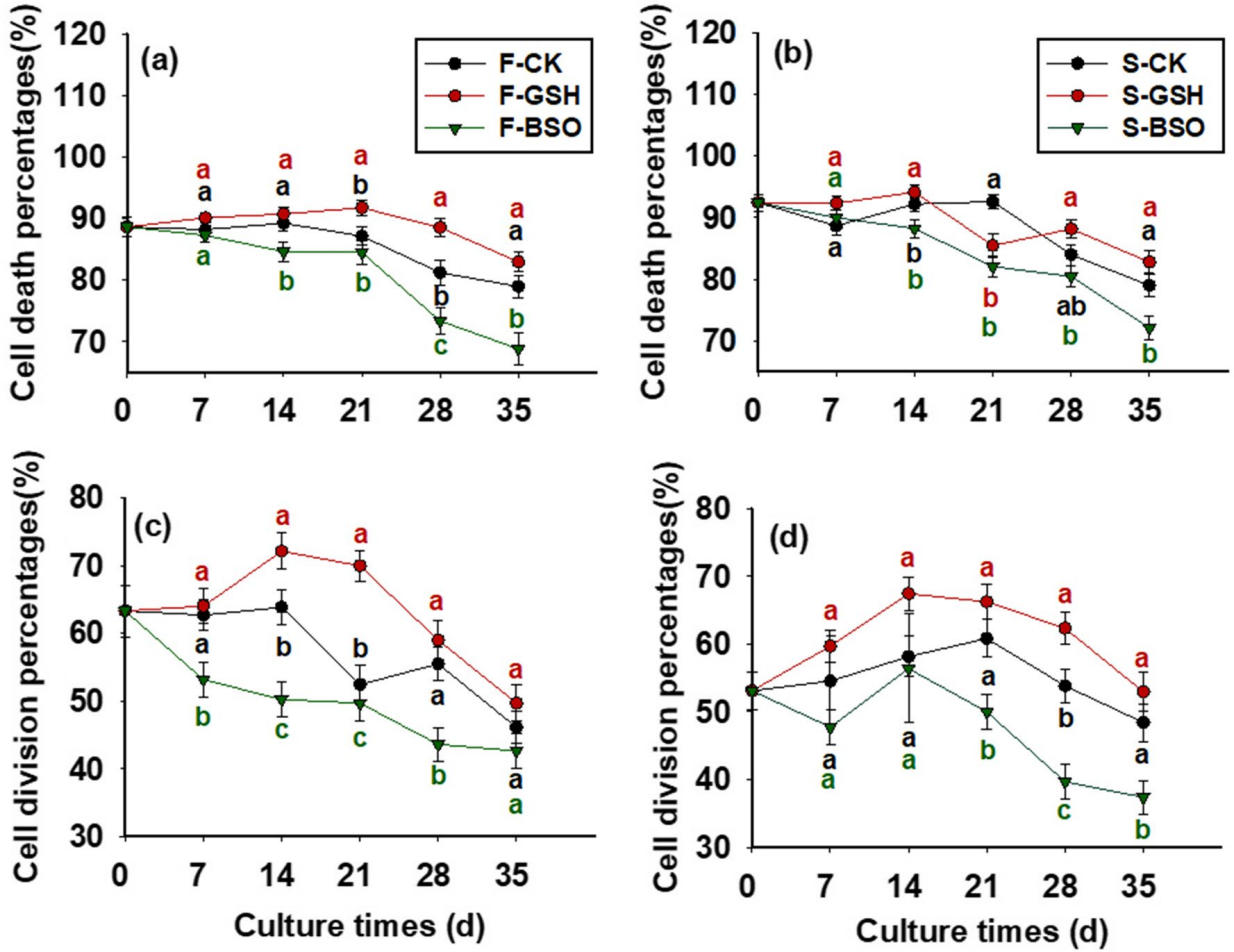
Effects of exogenous GSH and BSO on cell death and cell division percentages in two Korean pine cell lines. Note: F cell line control treatment (F-CK); F cell line exogenously supplemented with GSH (F-GSH); F cell line exogenously supplemented with BSO (F-BSO); S cell line control treatment (S-CK); S cell line exogenously supplemented with GSH (S-GSH); S cell line exogenously supplemented with BSO (S-BSO). ANOVA and Duncan's test were performed on the data (Mean ± se) in the figure. Different lowercase letters at the same little figure indicate significant differences (*p* < 0.05). Red lowercase letters indicate F-GSH and S-GSH treatment, black lowercase letters indicate F-CK and S-CK treatment, green lowercase letters indicate F-BSO and S-BSO treatment. The note of Figs. 3, 4 and 6 is the same as this figure.

The exogenous addition of GSH had a increasing effect on cell death percentages in the F cell line (Fig. 2a), and the cell death percentages of F-GSH gradually decreased (8.22% on day 21) from day 0 to 21 of the proliferation culture, and gradually increased to 17.00% from day 21 to 35. The exogenous addition of BSO could decrease the cell death percentages in the F cell line, and the cell death of F-BSO gradually increased from day 0 to 35 of the proliferation culture. The amount of cell death percentages gradually increased from day 0 to 35 of F-BSO proliferation culture, and the amount of cell death percentages was 31.14% on day 35. S and F cell lines showed a similar pattern (Fig. 2b).

### Effects of exogenous GSH and BSO on the percentage of division in EC cells with different proliferative potential

The exogenous addition of GSH showed a promoting effect on cell division in both F and S cell lines (Fig. 2c,d). The cell division frequency in F-CK treatment decreased from day 0 to 7 of proliferation culture, when day 35 decreased to 46.14% (Fig. 2c). The cell division frequency in F-GSH treatment gradually increased to 72.16% from day 0 to 14 of proliferation culture. The cell division frequency gradually reduced from day 14 to 35 (49.71% on day 35). The exogenous addition of BSO inhibited cell division in both F and S cell lines, and the cell division frequency gradually reduced from day 0 to 21, when day 35 decreased to 42.67% after F-BSO treatment.

### Effects of exogenous GSH and BSO on total protein (TP) content of EC cells with different proliferative potential

**Figure 3:**
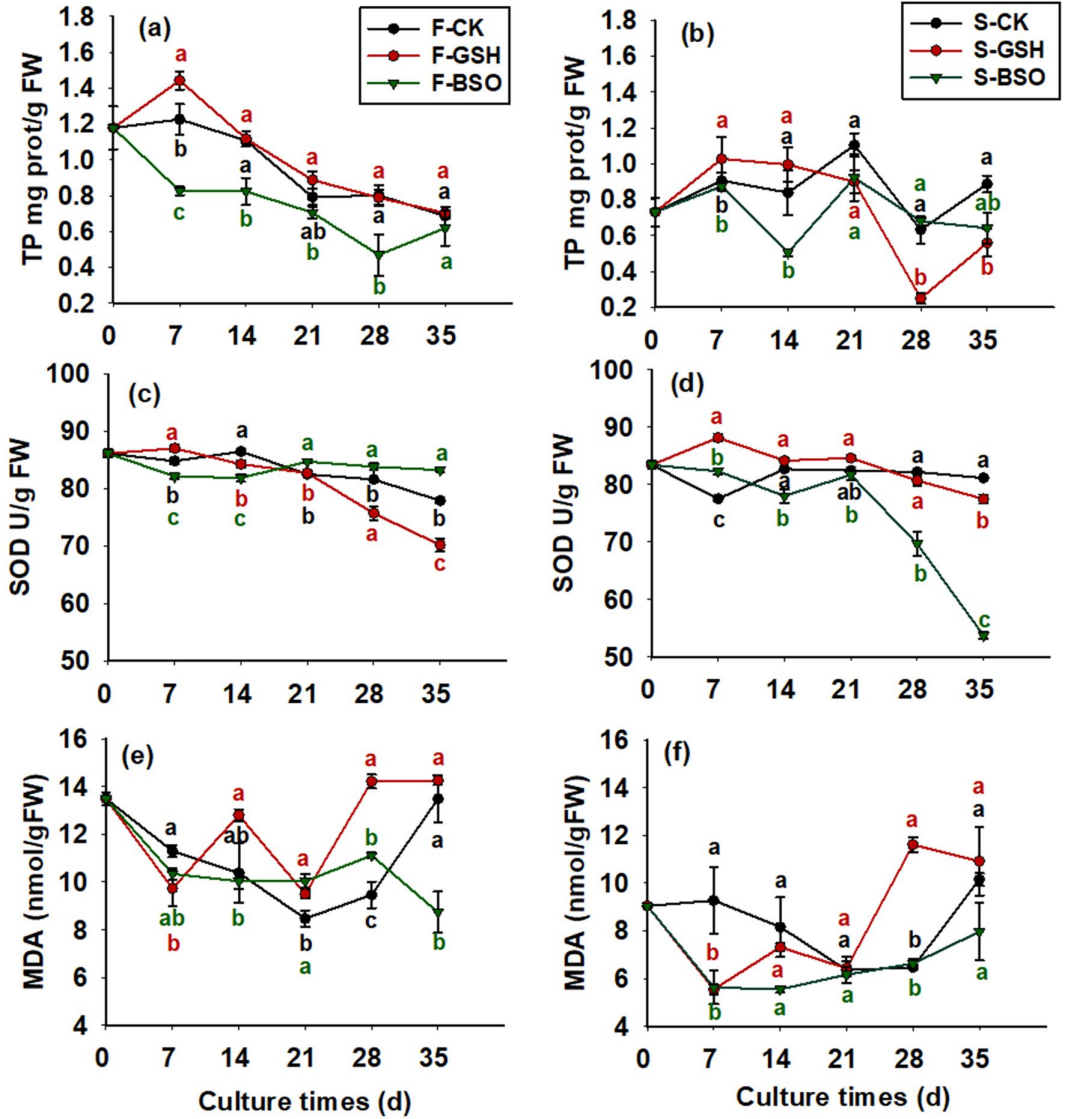
Effects of exogenous GSH and BSO on TP, SOD, MDA proliferation in two Korean pine cell lines.

Exogenous GSH increased intracellular TP content of F cell lines (Fig. 3a). Intracellular TP content of F-GSH increased to 1.44 mg prot/g FW on day 0–7 of proliferation culture and gradually decreased to 0.70 mg prot/g FW on days 7–35. Intracellular TP content of F-BSO decreased on days 0–28 of proliferation culture, decreasing to 0.69 mg prot/g FW on day 35. Intracellular TP content of F-BSO gradually decreased to 0.479 mg prot/g FW on days 0–28 and increased to 0.62 mg prot/g FW on days 28–35.

The exogenous addition of GSH promoted TP content of both cell lines. Compared with CK, the TP content of the F and S cell lines increased by 17.9% and 10.5% on day 7 of culture. During 7–35 days of S-CK proliferation culture, the TP content of cells first decreased (Fig. 3b), then increased and then decreased. On day 35, the content was 0.89 mg prot/g FW for S-CK, 0.56 mg prot/g FW for S-GSH, and 0.64 mg prot/g FW for S-BSO.

### Effects of exogenous GSH and BSO on SOD activity in EC cells with different proliferative potential

The effects of exogenous GSH and BSO on intracellular SOD activity of the two cell lines differed significantly (*p* < 0.05; Fig. 3). Exogenous GSH increased intracellular SOD activity (Fig. 3c), and on day 7 of culture, the SOD activity was 87.07 U/g FW for F-GSH treatment, 84.84 U/g FW for F-CK, and 82.18 U/g FW for F-BSO, which was 2.6% higher compared with F-CK. Intracellular SOD activity gradually decreased from day 0 to 35 in F-GSH proliferation culture (70.22 U/g FW on day 35). Exogenous BSO decreased intracellular SOD activity in both cell lines, and intracellular SOD activity gradually decreased from day 0 to 14 in F-BSO proliferation culture (81.91 U/g FW on day 14), and increased first and then decreased from day 14 to 35 (83.29 U/g FW on day 35).

On day 35 of proliferation culture, SOD activity for S-CK was 81.16 U/g FW (Fig. 3d), S-GSH was 77.47 U/g FW, and S-BSO was 53.74 U/g FW, and SOD activity for S-BSO was instead reduced by 51% compared with the F cell line. However, overall, it appeared that exogenous GSH increased intracellular SOD activity and exogenous BSO decreased intracellular SOD activity in both cell lines.

### Effects of exogenous GSH and BSO on MDA content of EC cells with different proliferative potential

The effects of exogenous GSH and BSO on intracellular MDA content of the two cell lines differed significantly (*p* < 0.05; Fig. 3). Exogenous GSH inhibited intracellular MDA content of F cell lines from day 0 to 14 (Fig. 3e), promoted it from day 14 to 28, and inhibited it from day 28 to 35. The exogenous addition of BSO increased intracellular MDA content, which decreased from day 0 to 7 in F-BSO proliferation culture (9.73 nmol/g FW on day 7), and increased from day 7 to 35 (14.73 nmol/g FW on day 35). Intracellular MDA content showed a trend of increasing first, decreasing, and then increasing (14.25 nmol/g FW on day 35).

Intracellular MDA content of F-CK cells was higher than that of S-CK cells during the culture period from day 0 to 35 (Fig. 3f). Intracellular MDA content for S-BSO showed an increasing trend from day 0 to 7 (9.27 nmol/g FW on day 7), and then decreased first and then increased from day 7 to 35 (10.15 nmol/g FW on day 35).

### Effects of exogenous GSH and BSO on CAT activity in EC cells with different proliferative potential

**Figure 4:**
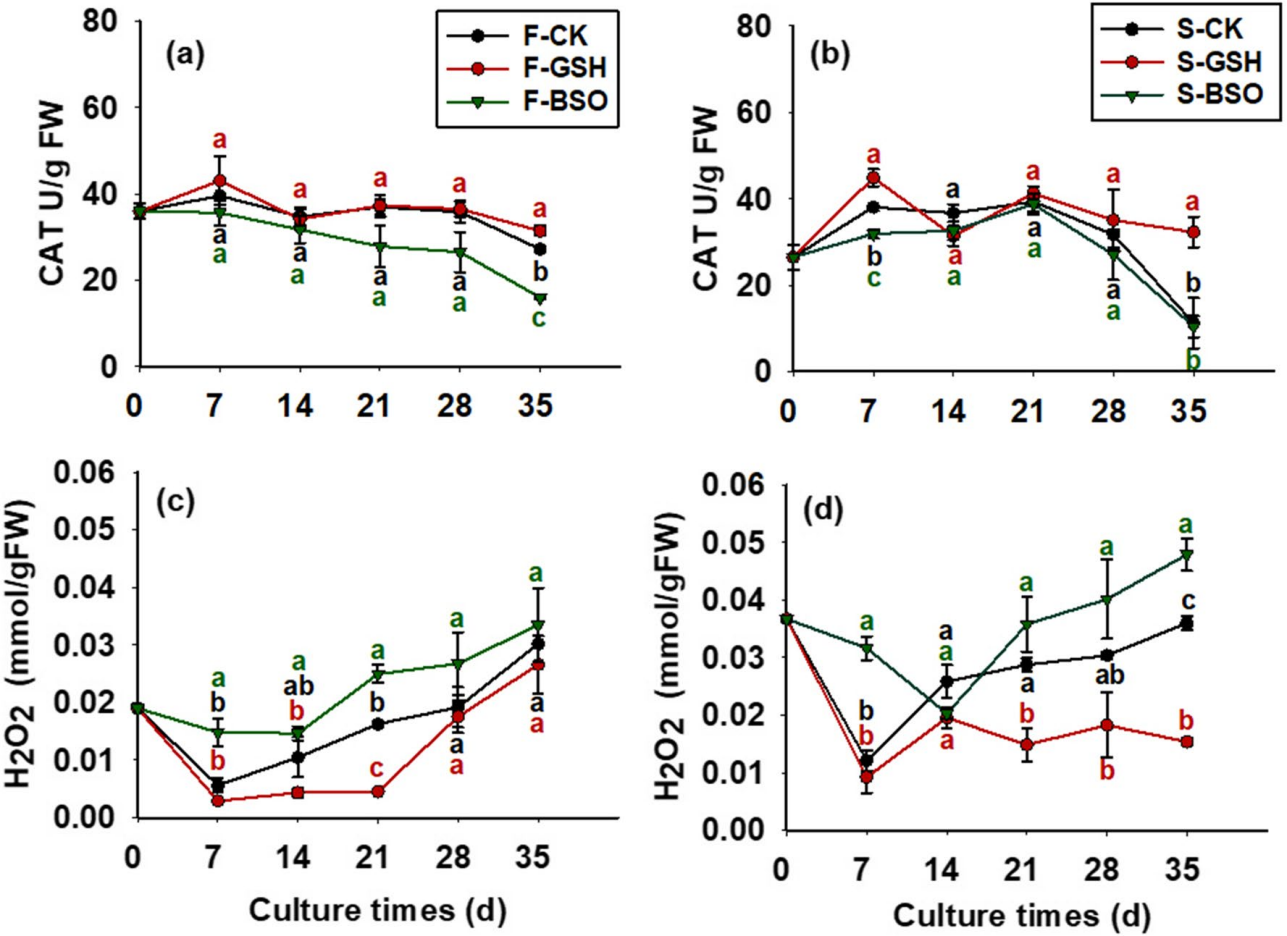
Effects of exogenous GSH and BSO on CAT and H_2_O_2_ in two Korean pine cell lines.

The effects of exogenous GSH and BSO on intracellular CAT activity in both cell lines were significantly different (*p* < 0.05; Fig. 4). The exogenous addition of GSH promoted intracellular CAT activity, which and intracellular CAT activity of F-GSH proliferation culture showed an increasing trend from day 0 to 7 (43.10 U/g FW on day 7; Fig. 4a), and decreased at first, increased, and then decreased from day 7 to 35, and CAT activity was 31.42 U/g FW on day 35. The exogenous addition of BSO reduced intracellular CAT activity of the F cell line. Intracellular CAT activity of F-BSO gradually decreased from day 0 to 35 of the culture period, and intracellular CAT activity on day 35 was 16.02 U/g FW (half of that of F-GSH).

In general, it appeared that the exogenous addition of BSO inhibited intracellular CAT activity in the S cell line. Compared with S-CK, the exogenous addition of GSH showed a positive effect on intracellular CAT activity in both cell lines except on day 14 of proliferation culture (Fig. 4b). CAT activity in F-CK cells was greater than that in S-CK cells throughout the proliferation period from day 0 to 35, with 35.98 U/g FW in F-CK cells on day 0 compared with only 26.50 U/g FW in S-CK cells.

### Effects of exogenous GSH and BSO on intracellular H_2_O_2_ content of EC cells with different proliferative potential

The effects of exogenous GSH and BSO on intracellular H_2_O_2_ content of the two cell lines differed significantly (*p* < 0.05; Fig. 4). Intracellular H_2_O_2_ content of F-CK cells tended to decrease from day 0 to 7 (0.006 mmol/g FW; Fig. 4c) and gradually increased to 0.030 mmol/g FW from day 7 to 35. Exogenous GSH inhibited intracellular H_2_O_2_ content of F cell lines. Intracellular H_2_O_2_ content of F-GSH proliferation culture tended to decrease from day 0 to 7 (0.003 mmol/g FW on day 7, half of CK) and gradually increased to 0.027 mmol/g FW from day 7 to 35 (close to CK). The exogenous addition of BSO promoted intracellular H_2_O_2_ content of F cell lines, and intracellular H_2_O_2_ content of F-BSO proliferation culture showed a gradual decrease from day 0 to 14 (0.015 mmol/g FW on day 14, more than twice that of CK), and gradually increased to 0.033 mmol/g FW (close to CK) from day 14 to 35.

Intracellular H_2_O_2_ content of S-CK cells was higher than that of F-CK during the culture period from day 0 to 35 (Fig. 4d), and intracellular H_2_O_2_ content of S-CK cells was 0.037 mmol/g FW on day 0 of proliferation culture, which was almost twice as high as that of F-CK (0.019 mmol/g FW for F-CK). Overall, it appeared that the exogenous addition of BSO had a promoting effect on intracellular H_2_O_2_ content of S cell lines, whereas the exogenous addition of GSH had an inhibitory effect on intracellular H_2_O_2_ content.

### Effects of exogenous GSH and BSO on in situ H_2_O_2_ production in EC cells

**Figure 5:**
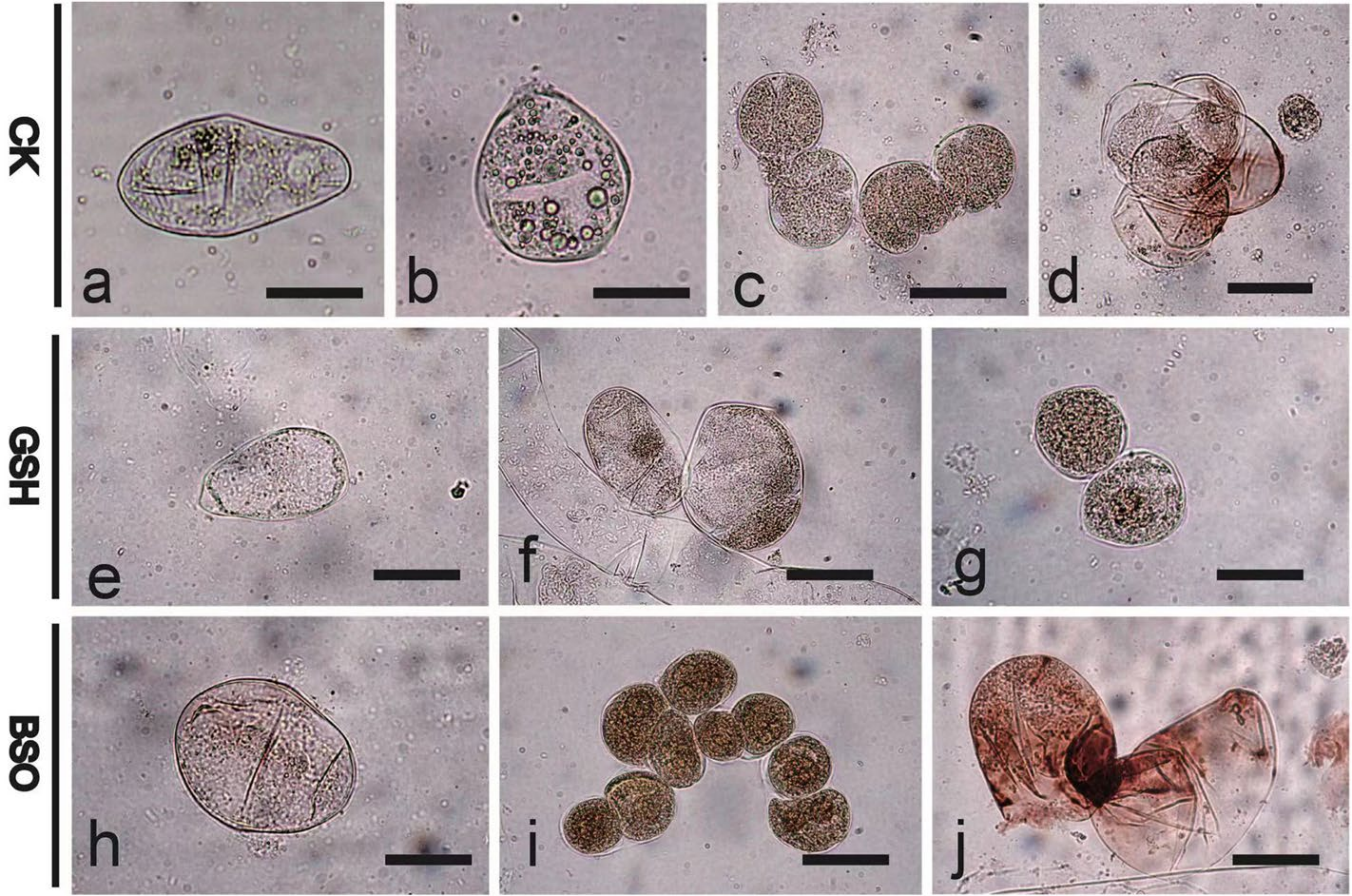
Effects of exogenous GSH and BSO on in situ H_2_O_2_ production in EC cells of Korean pine. (**a**, **e**, **i**) F-CK proliferation on day 0; (**b**) F-CK proliferation on day 7; (**c**) F-CK proliferation on day 21; (**d**) F-CK proliferation on day 35; (**f**) F-GSH proliferation on day 7; (**g**) F-GSH proliferation on day 21; (**h**) F-GSH proliferation on day 35; (**j**) F-BSO proliferation on day 7; (**k**) F-BSO proliferation on day 21; (**l**) F-BSO proliferation on day 35. Bars = 30 μm.

F-CK showed no obvious color production in EC cells on day 7 of proliferation culture (Fig. 5a), no obvious color production on day 7 (Fig. 5b), light yellow on day 21 (Fig. 5c), and dark yellow on day 35 (Fig. 5d).

F-GSH showed no obvious color production in cells on day 7 of proliferation culture (Fig. 5e), pale yellow on day 21 (Fig. 5f), and pale yellow on day 35 (Fig. 5g).

F-BSO treated cells were pale yellow on day 7 of proliferation culture (Fig. 5h), dark yellow on day 21 (Fig. 5i), and brown on day 35 (Fig. 5j).

The exogenous addition of GSH and BSO had a significant effect on intracellular H_2_O_2_ content of Korean pine EC cells. In situ intracellular H_2_O_2_ production showed a gradual increase with the increase in culture time. The overall trend of H_2_O_2_ production increased during the EC cell proliferation culture period from day 0 to 35 (day 7 < day 21 < day 35). Overall, the exogenous addition of GSH inhibited in situ intracellular H_2_O_2_ production, whereas the exogenous addition of BSO promoted in situ intracellular H_2_O_2_ production, and total in situ intracellular H_2_O_2_ production was in the order GSH < CK < BSO.

### Effects of exogenous GSH and BSO on NO content of EC cells with different proliferative potential

**Figure 6:**
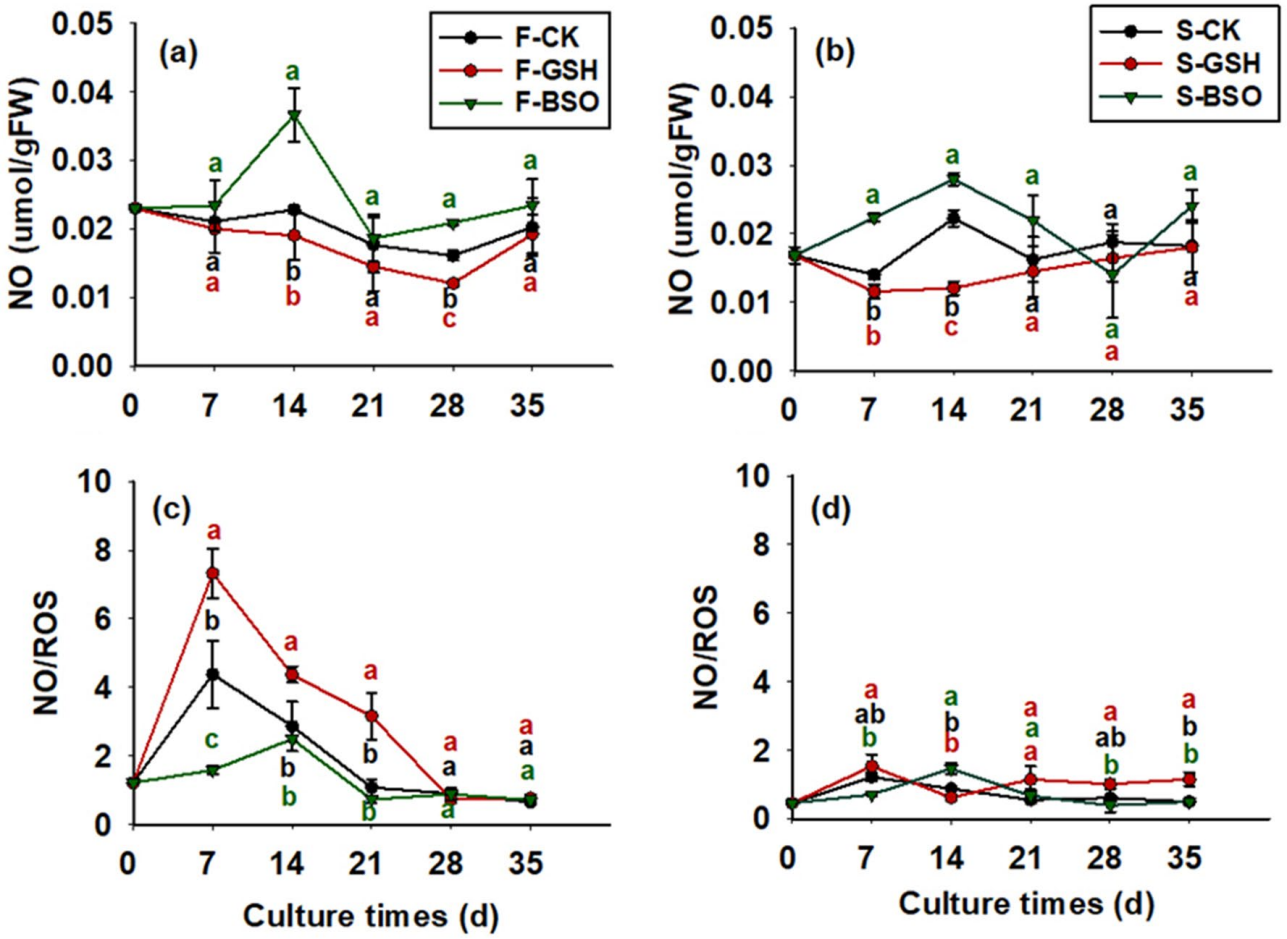
Effects of exogenous GSH and BSO on NO content of two Korean pine cell lines.

The effects of exogenous GSH and BSO on intracellular NO content of the two cell lines differed significantly (*p* < 0.05; Fig. 6). Intracellular NO content gradually decreased from day 0 to 28 in F-GSH proliferation culture (0.012 μmol/g FW on day 28; Fig. 6a) and increased from day 28 to 35 (0.019 μmol/g FW on day 35). It increased rapidly from day 0 to 14 in F-BSO proliferation culture to 0.037 μmol/g FW, decreased rapidly from day 14 to 21 (0.019 μmol/g FW on day 21), and gradually increased from day 21 to 35 and reached 0.023 μmol/g FW on day 35.

NO content of S-CK was lower than that of F-CK during the culture period from day 0 to 35 (Fig. 6a,b), and intracellular NO content was 0.017 μmol/g FW for S-CK and 0.023 μmol/g FW for F-CK on day 0. On the whole, it appeared that exogenous BSO had a promoting effect on intracellular NO content of F and S cell lines, and exogenous GSH had a depressant effect on intracellular NO content of both cell lines.

### Effects of exogenous GSH and BSO on the NO/ROS ratio in EC cells with different proliferative potential

The NO/ROS ratio in S-CK was lower than in F-CK during the culture period from day 0 to 35 (Fig. 6c,d), with an intracellular NO/ROS ratio of 0.46 in S-CK on day 0 (Fig. 6d) and 1.22 in F-CK. The effects of exogenous GSH and BSO on the intracellular NO/ROS ratio differed significantly (*p* < 0.05) between the two cell lines (Fig. 6c,d). The intracellular NO/ROS ratio in F-BSO proliferation culture increased rapidly from day 0 to 7 (7.32 on day 7) and gradually decreased from day 28 to 35 (0.76 on day 35). The intracellular NO/ROS ratio in F-BSO proliferation culture increased to 2.49 from day 0 to 14, and the intracellular NO/ROS ratio was lower from day 14 to 35 to 0.72 on day 35. In general, it appeared that exogenous BSO decreased the intracellular NO/ROS ratio in F and S cell lines, whereas exogenous GSH increased the intracellular NO/ROS ratio in both cell lines.

## Discussion

Conifer somatic embryogenesis is mostly indirect, which includes induction, proliferation, maturation, and plant regeneration^16^. EC proliferation is the key step of somatic embryogenesis, so the ability of EC proliferation and maturation is greatly important. The morphology and metabolites of spruce EC cells differ significantly with the exogenous addition of GSH, which could promote EC proliferation. Exogenous GSH could increase the number of early somatic embryos in Brazilian pine (*Araucaria angustifolia*) EC^17^. We found that the exogenous addition of GSH during Korean pine SE promoted EC cell proliferation, but the effects on its EC cell morphology and structure are unclear. In this study, the GSH content of Korean pine EC cells was changed by the exogenous addition of GSH and BSO. The exogenous addition of GSH increased EC cell proliferative potential, improved the state of EC cells. The result laid the foundation for the establishment of an efficient and stable technological system for somatic embryogenesis of Korean pine.

The GSH content of Korean pine EC cells was changed by the exogenous addition of GSH and BSO^12^. The exogenous addition of GSH had a promoting effect on EC cell proliferation and that of BSO had an inhibitory effect on EC cell proliferation compared with CK^18^. Nevertheless, the effect of GSH on EC cell division is unknown. Mitosis is the process by which eukaryotic cells divide to produce somatic cells. Because Korean pine is a eukaryote, its EC cells exhibit mitosis during proliferation. In this study, the cell division ratio of S-CK was smaller than that of F-CK. The exogenous addition of GSH increased the cell division ratio, whereas the exogenous addition of BSO decreased the cell division ratio. Therefore, it appears that GSH promoted EC cell proliferation in association with an increased percentage of cell division. ROS is an important regulator of cell division and differentiation. An increase in the ROS level leads to a decrease in the frequency of cell division levels^19^. Thus, it appears that exogenous GSH may promote Korean pine embryonic cell division by decreasing ROS levels.

Before an outbreak of ROS, some cells in the organism tend to protect others by actively dying, and this death is considered an important factor in the defense strategy of plants against pathogens that feed on living host tissue. A mass of ROS generation is accompanied by programmed cell death^20^. In this study, the exogenous addition of BSO increased cell death in both cell lines, this might be related to intracellular ROS metabolism, where exogenous BSO increased the ROS level and induced an increase in cell death, whereas when the exogenous addition of GSH decreased the intracellular ROS level, thus reducing cell death and facilitating cell proliferation. Whether cell death in this study was programmed cell death remains to be confirmed.

The organism provides the necessary energy for protein synthesis by consuming sugars. The conversion and accumulation between starch, soluble sugar, and soluble protein contribute to somatic embryogenesis^21^. Protein plays an important role in both conifer conidiophore development and somatic embryogenesis, and protein metabolism is dynamic in this process. In *Pinus bungeana*^22^, the content of soluble proteins was found to be higher in embryogenic callus than in non-embryonic callus. In *Picea mariana*, the addition of ABA increased the protein content^23^. Although TP content of both F and S cell lines showed a relatively consistent pattern, the TP content of F-CK was higher than that of S-CK. The addition of exogenous GSH increased intracellular TP content, whereas the addition of exogenous BSO decreased intracellular TP content. The proliferative capacity of F cell lines was higher than that of S cell lines, and exogenous GSH contributed to the increased proliferative capacity and TP content of Korean pine EC, which further indicated that the TP content was related to the proliferative capacity of EC, and the stronger the proliferative capacity, the higher the TP content. SOD is an important antioxidant enzyme that is essential for the scavenging of free radicals. It is also important for regulating ROS in organisms, which can reduce the damage caused by membrane lipid peroxidation^24^, and catalyzing the disproportionation reaction (2 O_2_^·−^ + 2H^+^  → O_2_ + H_2_O_2_) of O_2_^·−^ radicals^25^, thereby removing O_2_^·−^. The SOD level in an organism is a direct indicator of senescence and death. In contrast, MDA is an important production of membrane lipid peroxidation. The level of MDA can reflect the degree of membrane lipid peroxidation, thus indirectly reflecting the degree of membrane system damage and plant resilience. In the process of research, SOD often cooperates with MDA measurement, the level of SOD can reflect the organism's ability to scavenge ROS, while the level of MDA can reflect the degree of ROS damage to the organism, so the analysis of SOD and MDA together can help to better understand the status of intracellular ROS. The exogenous addition of ascorbic acid to tobacco cells during cell division reduced the intracellular MDA content and also accelerated the rate of cell division, which contributed to the reduction of cell membrane permeability and soluble protein content as well as the stability of cell membrane structure^26^.

Superoxide in plants can be converted to with SOD catalysis, and H_2_O_2_ needs to be scavenged by peroxidase. CAT mainly exists in peroxidase bodies in plant cells, which can not only catalyze the H_2_O_2_ reaction, but also oxidize other small molecules of toxic substances. H_2_O_2_, as an important form of ROS, can be scavenged by CAT^27,28^.In addition, some studies have shown that GSH is involved in controlling intracellular level of H_2_O_2_^29,30^. In this study, the level of H_2_O_2_ in F-GSH-treated cells decreased after exogenous addition of GSH and increased after exogenous addition of BSO. Therefore, the decrease in the percentage of cell death after exogenous addition of GSH may be related to the decrease in intracellular level of H_2_O_2_. ROS generation is accompanied by NO production^30–32^. NO is a key redox-active, small molecule involved in various aspects of plant growth and development^33^. A growing body of evidence suggests that NO and SNO are important mediators in plant cell death^34–36^. NO has a dual effect on plants, that is, low NO levels can help scavenge ROS and protect plants from oxidative damage by inducing the expression of genes related to antioxidant enzymes, whereas high NO levels interact with ROS to produce substances that damage the structure and function of the plant^37^. There are multiple interactions between NO and GSH. At the transcriptional level, genes stimulated by sodium nitroprusside (SNP) and nitrosoglutathione (GSNO) are involved in GSH synthesis, resulting in increased levels of total GSH in alfalfa (*Medicago sativa*) roots^38^. Therefore, most studies showed that SNP treatment increased total GSH content. An increase in NO content and H_2_O_2_ release was observed during suspension culture of OCD cells of *Populus alba*^39^. The synergistic effect of NO and ROS induced host cell death in the soybean cell suspension system^40^. In this study, the exogenous addition of BSO showed a promoting effect on intracellular NO content of F cell lines, whereas the exogenous addition of GSH showed an inhibitory effect on intracellular NO content, indicating that GSH is involved in the regulation of intracellular NO synthesis, whereas the exogenous addition of BSO withdrew or reduced the GSH level, prompting rapid intracellular NO production, which was similar to the findings of Vieira^17^.

## Conclusions

In summary, the exogenous addition of GSH reduced cell death and promoted cell division in both cell lines. Exogenous GSH had a promoting effect on intracellular ROS metabolism in EC cells of both cell lines of Korean pine, increased intracellular SOD and CAT activities, inhibited the production of intracellular H_2_O_2_, MDA, and NO, and increased the NO/ROS ratio. This study revealed the changes in ROS metabolism in the proliferation of Korean pine EC cells promoted by exogenous GSH, providing a theoretical basis for improving the proliferative potential of Korean pine EC cells and perfecting the plant regeneration systems for large-scale breeding.

## Materials and methods

### EC cell acquisition and proliferation culture

The plants used in this study complies with international guidelines. Full sibling family cones 1# were authorized to be collected from cooperative institution (Korean pine seed orchard of Lushuihe Forestry Bureau of Jilin Province) on July 1, 2018. Two cell lines (001#-001 and 001#-010) cultured after cryopreservation were used as test materials, they were all induced by 1# family. In this study, 001#-001 was abbreviated as F (Fast proliferative potential cell line is abbreviated as F), and 001#-010 was abbreviated as S (Slow proliferative potential cell line is abbreviated as S).

EC cell induction medium was mLV + Gelrite 4 g/L + l-glutamine 500 mg/L + sucrose 30 g/L + acid hydrolyzed casein 0.5 g/L + NAA 2 mg/L + 6-BA 1.5 mg/L.

EC cell proliferation medium was mLV + Gelrite 4 g/L + l-glutamine 0.5 g/L + acid-hydrolyzed casein 0.5 g/L + 2, 4-d 1 mg/L + 6-BA 0.5 mg/L + sucrose 30 g/L.

GSH (0.5 mmol/L) (GSH was dissolved in cool sterile water, the pH was adjusted to 5.8, filtered through a filter membrane (0.22-μm pore size), and sterilized before being added to the medium) or 0.5 mmol/L BSO (the medium was sterilized and cooled to about 55–60 °C, and added by filter sterilization using a filter membrane with a 0.22-μm pore size) was added to the F and S cell lines proliferation medium. The treatment without the addition of GSH or BSO was used as CK.

Samples were taken on days 0, 7, 14, 21, 28, and 35 of the proliferation culture, respectively, and their fresh weights, dry weights, cell death, cell division, TP, SOD, MDA, CAT, H_2_O_2_, in situ H_2_O_2_ and NO were measured. Exogenous GSH, BSO, and CK treatments of the F cell line are hereafter abbreviated as F-GSH, F-BSO, and F-CK, respectively.

### Morphology and internal structure of EC cells

EC cells treated with GSH, BSO, and CK for 15 days were observed for external morphology. The observation method was as follows: EC cells of the two cell lines after different treatments were observed and photographed under an optical microscope (OLYMPUS SZX 7, Japan, equipped with a Canon DS126271 camera, Japan).

EC cells from the two cell lines with different treatments were placed on the slides, stained with 0.1% Pan red staining solution for 10 min, covered with coverslips, and the coverslips were gently tapped with the flat end of a pencil to disperse EC cells evenly, which were immediately observed and photographed with an optical microscope (OLYMPUS CX 31, Japan, equipped with a Canon DS126271 camera, Japan).

### Cell death

Evans blue staining method was used to determine whether Korean pine EC cells were alive or dead. Cell viability was determined by a modified Pietrowska method^33^. A small amount of EC cells were stained with 1 mL of Evans blue staining solution (the solutions containing 3 mmol/L CaCl_2_, 0.6 mol/L mannitol, and 0.25% Evans blue) for 10 min. EC cells were fully dispersed by vigorous shaking and washed with 50 mL of distilled water for 30 min. A small amount of EC cells was placed on a glass slide and covered with a coverslip. The coverslip was gently tapped with the flat end of a pencil to evenly distribute EC cells on the glass slide, and the cells were immediately observed and photographed under the optical microscope. Dead cells (dark blue) and live cells (unstained) were counted. Two types of cells were observed under the microscope after Evans blue staining: unstained for live cells and dark blue for dead cells. At least 300 cells per treatment were assessed at each time point. The formula for calculating of cell death percantages is:

$$ {\text{Cell}}\;{\text{death}}\;{\text{percentages}}\left( \% \right) = \frac{{{\text{Dead}}\;{\text{cells}}}}{{\left( {{\text{Dead}}\;{\text{cells}} + {\text{Living}}\;{\text{cells}}} \right)}} \times 100 $$

### Percentage of cell division

The percentage of cell division was measured according to a modified method of Pasternak and Potters et al.^11,41^. A small amount of EC cells was stained with 0.2% methyl orange solution for 10 min and shaken vigorously to disperse the EC cells sufficiently. A small amount of EC cells was placed on a glass slide, covered with a coverslip, and the coverslip was gently tapped with the flat end of a pencil to distribute EC cells evenly on the slide. EC cells were immediately observed and photographed under the optical microscope. The effects of GSH and BSO treatment on cell division frequency were evaluated by determining the cell division frequency by microscopy.

### Total protein (TP), MDA, H_2_O_2_, and NO content as well as SOD and CAT activities

#### TP

TP content was determined by Nanjing Jiancheng kit (Nanjing Jiancheng Institute of Biological Engineering, Nanjing, China, TP A 045-2). The principle of detection is: protein molecules have –NH_3_^+^ group, when coomassie brilliant blue is added to protein standard solution or sample, the anion on coomassie brilliant blue combines with protein –NH_3_^+^, making the solution turn blue, and the protein content can be calculated by measuring the absorbance. Weigh 0.2 g EC accurately, add 1.8 ml normal saline to it, homogenize in ice bath, and centrifuge at 2500 rpm/min for 10 min. The supernatant was added to the reagent according to the kit method, and the spectrophotometer wavelength was 595 nm with double distilled water to adjust the zero, and the absorbance value of each tube was measured. For specific methods, see the instruction for the kit. Each treatment was repeated for 3 times.

#### SOD

SOD activity was measured using the Nanjing Jiancheng kit (Nanjing Jiancheng Institute of Biological Engineering, Nanjing, China, SOD A 001-3). The principle of detection is: WST-1 (2-(4-iodophenyl)-3-(4-nitrophenyl)-5-(2,4-disulfophenyl)-2H-tetrazolium salt, monosodium salt) is able to react with superoxide anion to form a water-soluble dye, so it can detect the content of SOD. The samples were accurately weighed to 0.2 g EC, homogenized in 1.2 mL phosphate buffer (pH 7.2) in ice bath, and centrifuged at 3500 rpm/min for 10 min at 4 °C. The supernatant was added to the reagents according to the kit method, and the absorbance values were read by a 450 nm microplate reader (BioTek Epoch, 190806A). For specific methods, see the instruction for the kit. 4 replicates for each treatment.

#### MDA

The content of MDA was determined by Nanjing Jiancheng kit (Nanjing Jiancheng Institute of Biological Engineering, Nanjing, China, MDA A 003-3-1). The detection principle is : malondialdehyde (MDA) in lipid peroxide degradation products can be condensed with thiobarbituric acid to form a product in red color, which has the maximum absorbance and emission at 532 nm. Because the substrate is thiobarbituric acid so this method is called TBA method. Weigh 0.2 g EC accurately, add 1.8 mL of reagent 5 application extract, homogenize in ice bath, centrifuge at 4000 rpm/min for 10 min, take the supernatant and add the reagent according to the method of the kit, put it in the microplate reader (wavelength 530 nm) to determine the absorbance value of each well. For specific methods, see the instruction for the kit. 3 replicates for each treatment.

#### CAT

The content of CAT was determined by Nanjing Jiancheng kit (Nanjing Jiancheng Institute of Biological Engineering, Nanjing, China, CAT A 007-1-1). The principle of the detection is: the reaction of decomposing H_2_O_2_ can be rapidly suspended by adding ammonium molybdate, and the remaining H_2_O_2_ interacts with ammonium molybdate to produce one kind of pale yellow complex, and the change amount can be measured at 405 nm to calculate the CAT activity. The content of CAT was determined by weighing 0.2 g EC accurately, adding 1.8 mL PBS buffer, homogenizing in ice bath, centrifuging at 2500 rpm/min for 10 min, taking the supernatant and adding reagents according to the kit method, spectrophotometer wavelength 405 nm, double distilled water for zero adjustment, and measuring the absorbance value of each tube. For specific methods, see the instruction for the kit. 3 replicates for each treatment.

#### H_2_O_2_

The content of H_2_O_2_ was determined by Nanjing Jiancheng kit (Nanjing Jiancheng Institute of Biological Engineering, Nanjing, China, H_2_O_2_ A 064-1-1). The principle of the detection is: H_2_O_2_ can interact with molybdenum acid to produce a complex and the amount of H_2_O_2_ can be calculated by measuring its production at 405 nm. Accurately weigh 0.2 g EC, add 1.8 mL PBS buffer, homogenize in ice bath, centrifuge at 10,000 rpm/min for 10 min, take the supernatant and add reagents according to the kit method, spectrophotometer wavelength 405 nm, double distilled water for zero adjustment. The absorbance values of each tube were measured, and the specific method was described in the kit instruction. 4 replicates for each treatment.

#### NO

The NO content was determined by Nanjing Jiancheng kit (Nanjing Jiancheng Institute of Biological Engineering, Nanjing, China, NO A 013-2-1). The principle of the detection is: NO with O_2_ and H_2_O generate nitrate and nitrite, and O_2_ and H_2_O can generate light red azo compound when meeting nitrate color agent. So the NO concentration can be indirectly measured through colorimetric detection. 0.4 g of EC was weighed accurately, 0.8 mL of pH 7 phosphate buffer was added, homogenized in an ice bath, and centrifuged at 3500 rpm/min for 10 min. The supernatant was added according to the method of the kit, and the absorbance of each well was measured at 550 nm by enzyme marker. The absorbance values of each well were measured by the microplate reader at 550 nm, and the specific method was described in the kit instruction. 4 replicates p for each treatment. NO/ROS ratio are calculated as the ratio of NO to H_2_O_2_ content.

### Effects of exogenous GSH and BSO on in situ H_2_O_2_ production in EC cells with different proliferative potential

In situ H_2_O_2_ production was determined according to the modified method of Orozco-Cárdenas and Ryan^34,42^. After incubating cells using 3,3′-Diaminobenzidine tetrahydrochloride (DAB) solution, the in situ formation of H_2_O_2_ was detected by forming brown precipitates. The specific method was as follows: EC cells were incubated with 1 mg/mL (pH 3.8) DAB at room temperature for 8 h, and stored in 75% ethanol until before observation (ethanol was pre-cooled at 4 °C). A small amount of EC cells was placed on the glass slide, covered with a coverslip, and the coverslip was gently tapped with the flat end of a pencil to distribute EC cells evenly on the slide, which was immediately observed and photographed under the optical microscope. The F cell line was used as the test material with the exogenous addition of GSH and BSO, and F cells without the addition of GSH and BSO were used as CK, and samples were taken on days 7, 21, and 35 of proliferation culture. At least 300 cells per treatment were assessed at each time point.

### Data statistics and analysis

Experimental data was analyzed using Excel 2003 (Microsoft Corp., Redmond, WA, USA). Means, NO and H_2_O_2_ content, and enzyme activities were calculated from the obtained data. One-way ANOVA and Duncan’s multiple comparisons were performed using SPSS 19 (IBM, Armonk, NY, USA). Graphs were plotted with Sigma Plot 12.0 (Systat, Chicago, IL, USA).

## Data Availability

All data generated or analysed during this study are included in this published article.
